# Chronic Urticaria Associated with Helicobacter pylori

**DOI:** 10.7759/cureus.4528

**Published:** 2019-04-23

**Authors:** Rajesh Essrani, Matthew Sullivan, Hiral Shah

**Affiliations:** 1 Internal Medicine, Lehigh Valley Health Network, Allentown, USA; 2 Gastroenterology, Lehigh Valley Health Network, Allentown, USA

**Keywords:** tetracycline, chronic urticaria, bismuth subsalicylate, helicobacter pylori, metronidazole, omeprazole

## Abstract

A 31-year-old male presented with a complaint of chronic pruritus with diffuse urticarial wheals for the past seven months. He underwent extensive workup and was found to have positive Helicobacter pylori (H. pylori) stool antigen test. He was treated with bismuth subsalicylate, metronidazole, tetracycline, and omeprazole for two weeks. Pruritus and urticarial wheals disappeared in four weeks after therapy was started.

## Introduction

Urticaria is a common skin disease characterized by widespread and transient wheals. It is classified as acute or chronic. Acute urticaria is defined as periodic outbreaks of urticarial lesions that resolve within six weeks, and chronic urticaria (CU) persists greater than six weeks [1]. The causes of CU are numerous; however, in at least 80%-90% of the patients, the etiology is undetermined [2-3]. The infection by Helicobacter pylori (H. pylori) has been the subject of investigation as a possible etiologic factor for CU in the last few years. H.pylori infection plays a role in the development of peptic ulcer disease, chronic active gastritis, and low-grade gastric mucosa-associated lymphoid tissue lymphoma and gastric malignancy [4-5]. We present an unusual case of CU in an adult patient with H. pylori infection and regression of chronic urticarial illness after treatment of H. pylori with bismuth-based quadruple therapy.

## Case presentation

**Figure 1:**
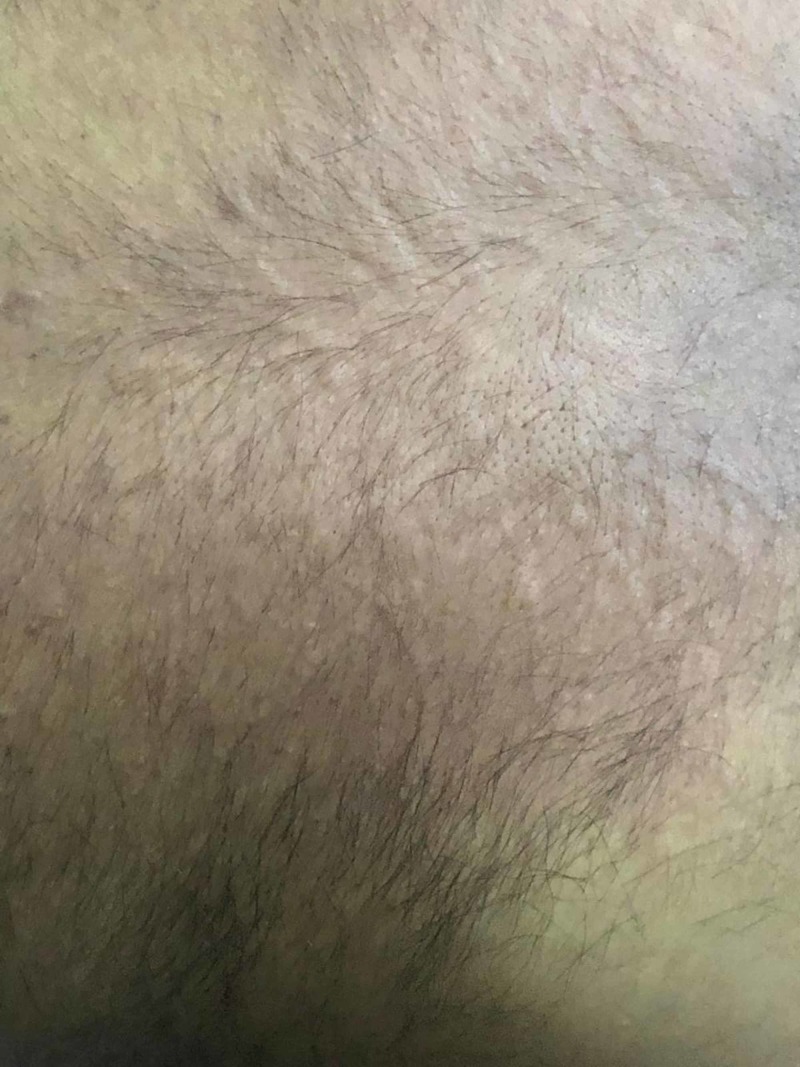
Urticarial wheals on the back

A 31-year-old male with a past medical history of mild intermittent asthma presented with a seven-month history of chronic pruritus with diffuse urticarial wheals (Figure 1).

**Figure 2:**
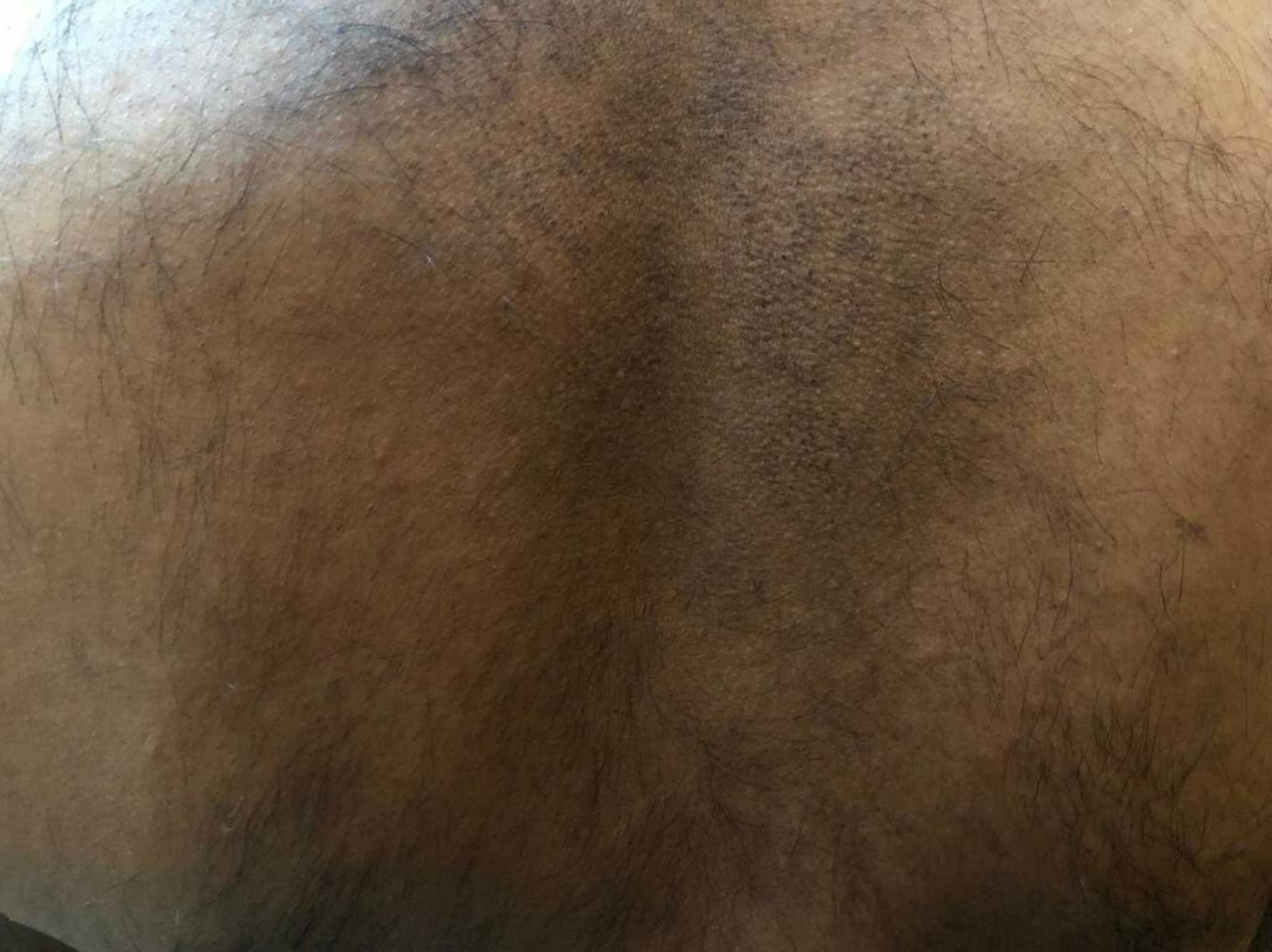
Disappearance of urticarial wheals after treatment

Allergy history for food, environment, and drugs was unremarkable. He underwent an extensive workup including complete blood count, basic metabolic panel, human immunodeficiency virus (HIV) testing, thyroid stimulating hormone, thyroid peroxidase antibodies, comprehensive stool panel, serum immunoglobulin E (IgE) level, and chest X-ray; all were unrevealing. He was initially treated empirically with cetirizine 5 mg daily without significant improvement. The cetirizine dose was subsequently increased to 10 mg with only minimal improvement. Ranitidine 150 mg twice daily was added but without much relief. A few weeks later, he complained of new onset of epigastric pain and was subsequently tested for H. pylori by stool antigen which resulted as positive. He was treated with bismuth subsalicylate, metronidazole, tetracycline, and omeprazole for two weeks. Pruritus and urticarial wheals disappeared four weeks after therapy was started (Figure 2).

Repeat stool H. pylori was performed eight weeks after completing antibiotics and off omeprazole and confirmed eradication. The patient has had no recurrence of urticaria following treatment.

## Discussion

CU is defined by the presence of urticaria, angioedema, or both for six weeks or longer. The clinical manifestations of CU are typically limited to the skin, but sometimes systemic symptoms are seen [6-7]. CU is associated with various autoimmune disorders such as thyroid disorders, celiac disease, Sjogren syndrome, systemic lupus erythematosus, and type 1 diabetes mellitus. First line treatment of CU is the H1 anti-histamines, and as the second line, corticosteroids, leukotriene antagonists, H2 anti-histamines, immunosuppressants, monoclonal antibodies, and intravenous human immunoglobulin [8-9].

H. pylori lives in the stomach and is a leading cause of peptic ulcer disease. H. pylori has also been linked to a variety of conditions which can affect the skin such as CU, rosacea, psoriasis, Henoch-Schönlein purpura, Sjögren syndrome, systemic sclerosis, generalized pruritus (itch), atopic dermatitis, and aphthous ulceration [10-11].

Multiple studies have shown an association between CU and H. pylori infection. It is thought that H. pylori increases the permeability of the stomach lining, therefore, increasing the exposure to allergens in the gastrointestinal tract. Also, the immune response to H. pylori yields antibodies that may stimulate the release of histamine in the skin [12]. IgE-containing cells in the gastrointestinal tract seem to be the culprit, but there is limited proof for H. pylori-specific IgE. Thus, the likelihood that patients with urticaria develop specific IgE against H. pylori is an appealing pathogenic explanation that likely has not been confirmed yet [10].

Shakouru et al. [13] evaluated 19 studies, 17 observa­tional, and two double-blinded, randomized, controlled clinical trials, and observed that 10 of these studies showed a beneficial impact of H. pylori eradication in the resolution of the symptoms of CU.

Endoscopic and non-endoscopic methods can establish the diagnosis of H. pylori infection. The non-endoscop­ic, less invasive techniques, include serologic testing, labeled urea breath test, and the monoclonal antibody-based H. pylori stool antigen test. The endoscopic tests, performed on gastric biopsy specimens obtained during upper endoscopy, are the rapid urease test, histopathology, and culture [14].

Choosing the initial regimen to treat H. pylori should be guided by the presence of risk factors for regional antibiotic resistance patterns and eradication rates. H. pylori should be treated for 14 days. Risk factors for macrolide resistance include prior exposure to macrolide therapy for any reason and high local clarithromycin resistance rates (≥ 15%) or eradication rates with clarithromycin-based triple treatment ≤ 85%. Initial treatment options include quadruple bismuth therapy containing proton pump inhibitor (PPI), bismuth, metronidazole, and tetracycline (PBMT), and concomitant non-bismuth quadruple treatment comprising PPI, amoxicillin, metronidazole, and clarithromycin (PAMC). Patients without risk factors for macrolide resistance should be treated with triple therapy containing clarithromycin, amoxicillin, and PPI (CAP) [15-17]. Amoxicillin can be substituted with metronidazole only in penicillin-allergic individuals [16-18]. Levofloxacin-based triple therapy containing levofloaxacin, amoxillin and PPI (LAP) and sequential non-bismuth quadruple therapy (PPI, amoxicillin followed by PPI, metronidazole, and clarithromycin) shouldn’t be used for first-line treatment [16].

Approximately 20% of patients fail an initial attempt at H. pylori eradication. In patients with persistent H. pylori infection, antibiotic therapy should be guided by the patient’s initial treatment regimen, the use of other antibiotics, and the presence of relevant antibiotic allergies. Antibiotics included in the initial regimen should generally be avoided when prescribing a subsequent regimen. PBMT and LAP can be used in patients with no previous metronidazole exposure. If levofloxacin therapy fails, then PBMT is the next option even if previously exposed to metronidazole. The use of rifabutin-containing regimens (rifabutin, PPI, amoxicillin) should be used in patients who have failed to respond to at least three prior options. There is insufficient data to support non-bismuth quadruple therapy and PAMC for rescue therapy.

Eradication should be confirmed in all patients treated for H. pylori because of increasing antibiotic resistance [15,18]. Confirmation of elimination can be established with a urea breath test, stool antigen testing, or endoscopy-based testing. Tests to confirm eradication should be performed at least four weeks after completion of antibiotic treatment [15]. PPIs should be held for one to two weeks before testing to reduce false-negative results. Serologic testing should not be performed to confirm eradication as patients may continue to have circulating antibodies even after eradication.

## Conclusions

CU and dyspepsia are associated with H. pylori and its presence can be tested for by using an affordable, noninvasive, sensitive and specific test such as H. pylori stool antigen. H. pylori should be treated for 14 days with appropriate therapy and eradication should be confirmed. The case demonstrates that H. pylori should be included in the diagnostic workup of chronic urticaria, especially in patients without response to habitual treatment for chronic urticaria or those with concurrent gastrointestinal symptoms.

## Appendices

This article has been published as a poster and can be found on the following website - https://www.eventscribe.com/2018/ACG/ajaxcalls/PosterInfo.asp?efp=RFNSWFFHSFY2NDI0&PosterID=160190
